# Tumor volume is an independent prognostic indicator of local control in nasopharyngeal carcinoma patients treated with intensity-modulated radiotherapy

**DOI:** 10.1186/1748-717X-8-208

**Published:** 2013-09-05

**Authors:** Mei Feng, Weidong Wang, Zixuan Fan, Binyu Fu, Jie Li, Shichuan Zhang, Jinyi Lang

**Affiliations:** 1 Department of Radiation Oncology, Sichuan Cancer Hospital, No. 55, Section 4, South Renmin Rd, Chengdu, Sichuan, People's Republic of China

**Keywords:** Primary tumor volume, Primary nodal volume, Nasopharyngeal carcinoma, Prognostic factor

## Abstract

**Background:**

To retrospectively analyze whether primary tumor volume and primary nodal volume could be considered independent prognostic factors for nasopharyngeal carcinoma treated with intensity-modulated radiation therapy.

**Methods:**

Three hundred sixty-three consecutive nasopharyngeal carcinoma (NPC) patients who were stage I-IVa+b and treated with intensity-modulated radiotherapy (IMRT) in our center from October 2003 to October 2005 were analyzed retrospectively. The predictive ability of gender, age, T and N stage, combined chemotherapy, primary tumor and nodal volume for the 5-year local control (LC), distant-metastasis free survival (DMFS) and overall survival (OS) rate were investigated. Primary tumor and nodal volume were measured based on registration of magnetic resonance imaging (MRI) with contrast-enhanced computed tomography (CT) images. The Kaplan–Meier method was used for survival analysis, the log-rank test was used for univariate analyses and the Cox proportional hazard model was used for multivariate prognostic analyses.

**Results:**

The mean value of primary tumor and nodal volume were 31.5 ml and 9.7 ml. The primary tumor and nodal volume were respectively divided into four groups for analysis (primary tumor volume: TV1≤20 ml, 20<TV2≤30 ml, 30<TV3≤40 ml, TV4>40 ml; primay nodal volume: NV1≤5 ml, 5<NV2≤10 ml, 10<NV3≤20 ml, NV4>20 ml). In univariate analysis, the 5-year LC and DMFS rate for TV4 was significantly decreased compared to the other groups (LC: *p*<0.001, DMFS: *p*=0.001), the 5-year OS rate for TV3 and TV4 were significantly decreased compared to other two subgroups (*p*=0.002) and the 5-year regional control (RC), DMFS and OS rate for NV3 and NV4 were significantly less than NV1 and NV2 (RC: *p*=0.002, DMFS: *p*=0.01, OS: *p*=0.014). Multivariate analysis showed that TV>40 ml was an adverse prognostic factor for the 5-year local regional control (LRC) rate (RR 2.454, *p*=0.002). Primary nodal volume had no statistical significance in predicting 5-year LRC, DMFS and OS rate in multivariate analysis.

**Conclusions:**

Primary tumor volume could predict LRC rate of NPC patients, and the primary tumor volume of 40 ml may be the cut-off. Primary nodal volume may have predictive significance, but more data are needed. These factors should be considered in the TNM staging system of NPC for better estimates of prognosis.

## Background

NPC is a cancer of the nasopharyngeal cavity that has a dramatic geographic and ethnic distribution. In some areas of China, the incidence is as high as 30/100,000. It is estimated that there are more than 50,000 newly diagnosed cases in China and 65,000 worldwide each year [1]. Radiotherapy, especially intensity modified conformal radiotherapy, is the major therapeutic approach for NPC [2]. Prognostic factors for nasopharyngeal cancer have been well-documented by several groups. T/N and clinical stage, chemotherapy, radiation interruptions and hemoglobin level are considered important prognostic factors for NPC patients [3]. In the current study, we include tumor volume as a candidate factor for predicting treatment outcome of NPC treated in our hospital. It was first proposed by Fletcher that tumor volume might indicate the number of tumor clonogens that should be removed [4]. The volume of the primary tumor varies in the T classification, which suggests a possible effect of tumor volume on survival after radiotherapy. Indeed, tumor volume is included in the staging systems of many types of tumors to predict prognosis. For example, primary tumor volume is considered in the lung cancer staging system. Larger lung tumor volume might indicate poorer survival rates [5]. The International Union Against Cancer (UICC) and the American Joint Committee of Cancer (AJCC) staging systems are widely used for NPC patients. Anatomic location of the lesion and cranial nerve involvement are the critical staging factors in this system. However, discussions on the prognostic and staging value of primary tumor and nodal volume in NPC are notably limited, which warranted the current study. Most studies used CT images to delineate and measure the primary tumor and nodal volume. In the current study, we used MRI to accurately evaluate the invasion of the carcinoma. The tumor volumes derived from MRI registration, as well as other important clinical parameters, was carefully evaluated for its power to predict treatment results.

## Methods

### Patient selection

From October 2003 to October 2005, a total of 447 NPC patients treated with IMRT in the Sichuan Cancer Hospital were retrospectively analyzed. The study was approved by the ethics committee of Sichuan Cancer Hospital. Sixteen patients were excluded because of distant metastases at the time of initial diagnosis. Among the remaining 431 patients, who were stage I-IVa+b, only 386 patients received a MRI of the head and neck at beginning of treatment. Ultimately, 363 cases had complete medical information and entered the retrospective analysis. All patients received complete physical examinations, endoscopy, CT and MRI of the head and neck, chest radiography, bone emission computed tomography and dental assessments before treatment. They received the nasopharyngeal neoplasm biopsy to confirm the NPC diagnosis pathologically. They were staged according to the UICC 2002 staging system. The characteristics and distribution of the patients are listed in Table 1.

**Table 1:** Clinical characteristic of 363 NPC patients

**Characteristics**	**N (%)**
Age	
Median age 48 years	
(range 16–78 year)	
≤48	276(76.0%)
>48	87(24.0%)
Gender	
Male	285(78.5%)
Female	78 (21.5%)
Histopathology(WHO)	
Type I	17(4.7%)
Type II	216(59.5%)
Type III	130(35.8%)
T-classification	
T1	49(13.5%)
T2	116(31.9%)
T3	125(34.4%)
T4	73(20.2%)
N-classification	
N0	44(12.1%)
N1	166(45.7%)
N2	121(33.3%)
N3	32(8.9%)
Clinical stage	
I	16(4.4%)
II	94(25.9%)
III	165(45.4%)
IV	88(24.3%)
Chemotherapy	
YES	270(74.4%)
NO	93(25.6%)

### Volume measurement

All patients were placed in the supine position on a Medtec positioning system with their head, neck and shoulders fixed and a 2.0 cm cork in their mouth. Each patient underwent a localized, contrast-enhanced CT scan, with the cranial vertex as the upper limit and 2 cm below the inferior margin of the clavicle head as the lower limit. MRI was performed with a 1.5T system (Magnetom Avanto, Siemens, Germany) with a standard clinical imaging protocol. T1-weighted fast spin-echo images in the axial planes and T2-weighted fast spin-echo fat- suppressed images in the axial plane and coronal planes were obtained before the injection of contrast material. After intravenous administration of gadopentetate dimeglumine (Schering, Germany) at a dose of 0.1 mmol per kilogram of body weight, axial and sagittal T1-weighted fat-suppressed spin-echo sequences were performed sequentially. The scanning layer was 3.0 mm, and the layer interval was 2.5 mm. Registration of MRI with planning CT images was performed for all patients for accurate delineation of tumor volumes and critical structures. The primary nasopharyngeal tumor and neck positive nodes were outlined based on CT and MRI registration images. The retropharyngeal nodes were included in the primary nasopharyngeal tumor. Positive nodes in the neck were diagnosed according to the UICC2002 staging system. All images were evaluated by two head and neck clinicians. Radiotherapy planning was designed and optimized using the CORVUS 3.4-4.2 inverse treatment planning system (Corvus, Nomos Corporation, Sewickley, PA, USA). The volumes of primary tumor and nodes were calculated by the summation-of-area technique in the system, which multiplied the entire areas by the image reconstruction interval.

### Treatment

#### Radiotherapy

A full course of IMRT was used in all patients. According to the definitions of the International Commission on Radiation Units and Measurements (ICRU) 50 and 62, the target volumes were outlined in each layer of the CT images on an IMRT workstation. The gross tumor target of nasopharynx (GTVnx) and right/left lymph nodes (GTVln-R/L) were outlined based on the borders of the nasopharyngeal tumor and lymph nodes as shown by CT and MRI. High risk clinical target volume (CTV1) includes the GTVnx with a 5 to 10 mm margin and high-risk structures. A smaller margin was used for the gross nasopharyngeal tumor where it was adjacent to critical neurologic structures. Low risk clinical target volume (CTV2) covered CTV1 and was designed for potentially involved regions including the nasopharyngeal cavity, maxillary sinus, pterygopalatine fossa, posterior ethmoid sinus, parapharyngeal space, skull base, anterior third of clivus, inferior sphenoid sinus and cavernous sinus et al. CTVln covered lymphatic drainage regions (including the bilateral retropharyngeal nodes at levels II, III and VA). Radiotherapy planning was designed and optimized using the CORVUS 3.4-4.2 inverse treatment planning system. The prescribed doses of each target area were as follows: 66–76 Gy for GTVnx, 60–70 Gy for GTVlnR/L, 60–66 Gy for CTV1, 54–60 Gy for CTV2 and 50–54 Gy for CTVln. Each was divided into 30–33 deliveries. The dose limits for each normal organ were set according to the Radiation Therapy Oncology Group protocol 0225 (RTOG0225). The prescribed dose encompassed at least 95% of the target volume, no greater than 1% of nasopharynx gross target volume could receive ≤93% of the prescribed dose and the maximum dose of the treatment plan was inside the target volume. The IMRT plan was implemented through dynamic intensity-modulated coplanar arc irradiation using a multileaf collimator (Nomos mimic). All of the patients received radiation in the lymph node drainage areas in the lower neck using ^60^Co split-field techniques or 6 MV X-ray split-beam techniques with a prescription dose of 46-50 Gy.

#### Chemotherapy

Of the 363 patients, 93 patients received radiotherapy only, while 270 received IMRT combined with chemotherapy. Among these 270 patients, 57 received neoadjuvant and concurrent chemotherapy, 116 patients received concurrent chemotherapy, and the remaining 97 received concurrent-adjuvant chemotherapy. The patients who received neoadjuvant chemotherapy meant they received 2 to 3 cycles of cisplatin-based chemotherapy every 3 weeks before radiation. After completion of radiation, 1 to 2 cycle of adjuvant chemotherapy was given to the patients who had residual disease. The neoadjuvant and adjuvant chemotherapy protocol was paclitaxel 75 mg/m^2^ d1 + cisplatin 30 mg/m^2^ d1-3 or cisplatin 30 mg/m^2^ d1-3 + 5-Fu 750 mg/m^2^ d1-5. The concurrent chemotherapy protocol was cisplatin 30 mg/m^2^ d1-3 every 3 weeks for 2 to 3 cycle.

#### Follow-up

After completion of radiation and chemotherapy, patients were followed-up every 3 months in the first year, every 6 months in the second year and then every 12 months in the following 3 years. The follow-up period started from the date of diagnosis and ended on either the date of death or the date of the last follow-up. Local failure was defined as the recurrence at the nasopharyngeal cavity. Regional failure was defined as the recurrence of regional lymph nodes. Metastases to any site beyond the primary tumor and lymph nodes were defined as distant failure.

#### Statistical analysis

Statistical analysis was carried out using SPSS software (IBM company, version 19), and the survival rate was calculated using the Kaplan-Meier method. The log-rank test was used for univariate analyses of prognostic factors, and the Cox proportional hazard model was used for independent multivariate prognostic analyses. *P* < 0.05 was considered statistically significant.

## Results

### The volume statistics of primary tumor and nodes

The primary tumor and nodal volume were automatically measured by the CORVUS 3.4-4.2 inverse treatment planning system in 363 NPC patients. The mean value of primary tumor and nodal volume were 31.5 (range 1.8 ml-112.4 ml) and 9.7 (range 1.3-82.6 ml) respectively. The mean value of primary tumor volume in T1, T2, T3 and T4 groups was 12.1 ml, 21.5 ml, 31.6 ml and 56.4 ml. The range of primary tumor volume was 1.8-20.6 ml in T1 group, 4.3-67.8 ml in T2 group, 8.6-89.8 ml in T3 group, and 23.7-92.3 ml in T4 group. The patients were respectively divided into subgroups based on their primary tumor and nodal volume. The four subgroups for primary tumor volume were TV1≤20 ml, 20 ml<TV2≤30 ml, 30<TV3≤40 ml and TV4>40 ml; the four subgroups for primary nodal volume were NV1≤5 ml, 5 ml <NV2≤10 ml, 10 ml <NV3≤15 ml and NV4 >15 ml.

### Treatment outcome

The median follow-up time was 63 months (range 9–82 months) for all patients. The 5-year LC, RC, DMFS and OS rate were 92.2%, 91.0%, 75.2% and 79.3%, respectively. Forty-three cases experienced local failure, 43 cases had regional failure and 93 cases had distant metastases. There were 82 deaths.

### Univariate analysis

The UICC2002 staging system was used in all enrolled patients. The univariate analysis results for T/N stage are shown in Table 2. For the T stage, the 5-year LC rate was not significantly different (*p*=0.355). The 5-year DMFS and OS rate for T3/T4 groups were lower than T1/T2 groups (DMFS: T1 vs. T2, *p*=0.392; T3 vs. T4, *p*=0.861; T2 vs. T3, *p*=0.024. OS: T1 vs. T2, *p*=0.129; T3 vs. T4, *p*=0.684; T2 vs. T3, *p*=0.015). The 5-year RC rate was not significantly different between N stages (*p*=0.105). N3/4 groups had lower 5-year OS and DMFS rate than N1/2 groups (DMFS: N1 vs. N2, *p*=0.592; N3 vs. N4, *p*=0.152; N2 vs. N3, *p*=0.019. OS: N1 vs. N2, *p*=0.608; N3 vs. N4, *p*=0.537; N2 vs. N3, *p*=0.005).

**Table 2:** Univariate prognostic analysis of 363 NPC patients for T stage and N stage

**Prognostic factors**	**LC**		**RC**		**DMFS**		**OS**	
	**%**	**p**	**%**	**p**	**%**	**p**	**%**	**p**
T stage		0.355				0.014		0.002
T1	93.7				84.8		90.1	
T2	90.6				82.5		88.4	
T3	85.6				68.6		78.3	
T4	84.3				65.5		74.5	
N stage				0.105		0.003		0.005
N0			92.5		83.7		90.0	
N1			90.8		80.1		88.3	
N2			86.6		70.9		80.1	
N3			83.4		66.5		73.4	

**Figure 1:**
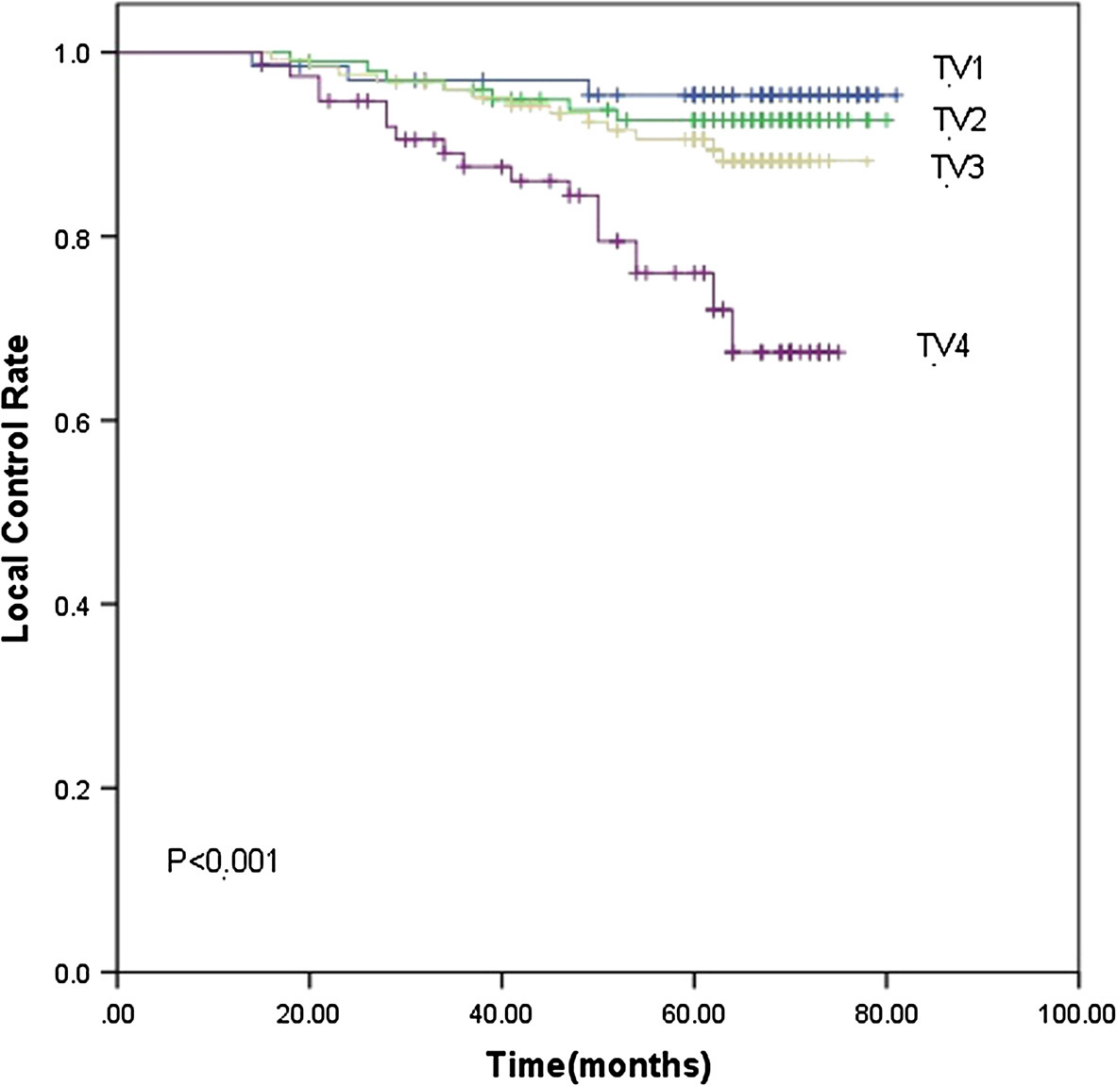
**Kaplan-Meier curves showing the 5-year LC rate in TV1, TV2, TV3 and TV4 groups.** There was a significant difference between the groups (*p*<0.001). The statistical analyses between each pair of groups were as follows: TV1 vs. TV2, *p*=0.512; TV1 vs. TV3, *p*=0.161; TV1 vs. TV4, *p*=0.000; TV2 vs. TV3, *p*=0.365; TV2 vs. TV4, *p*=0.000; and TV3 vs. TV4, *p*=0.001.

**Figure 2:**
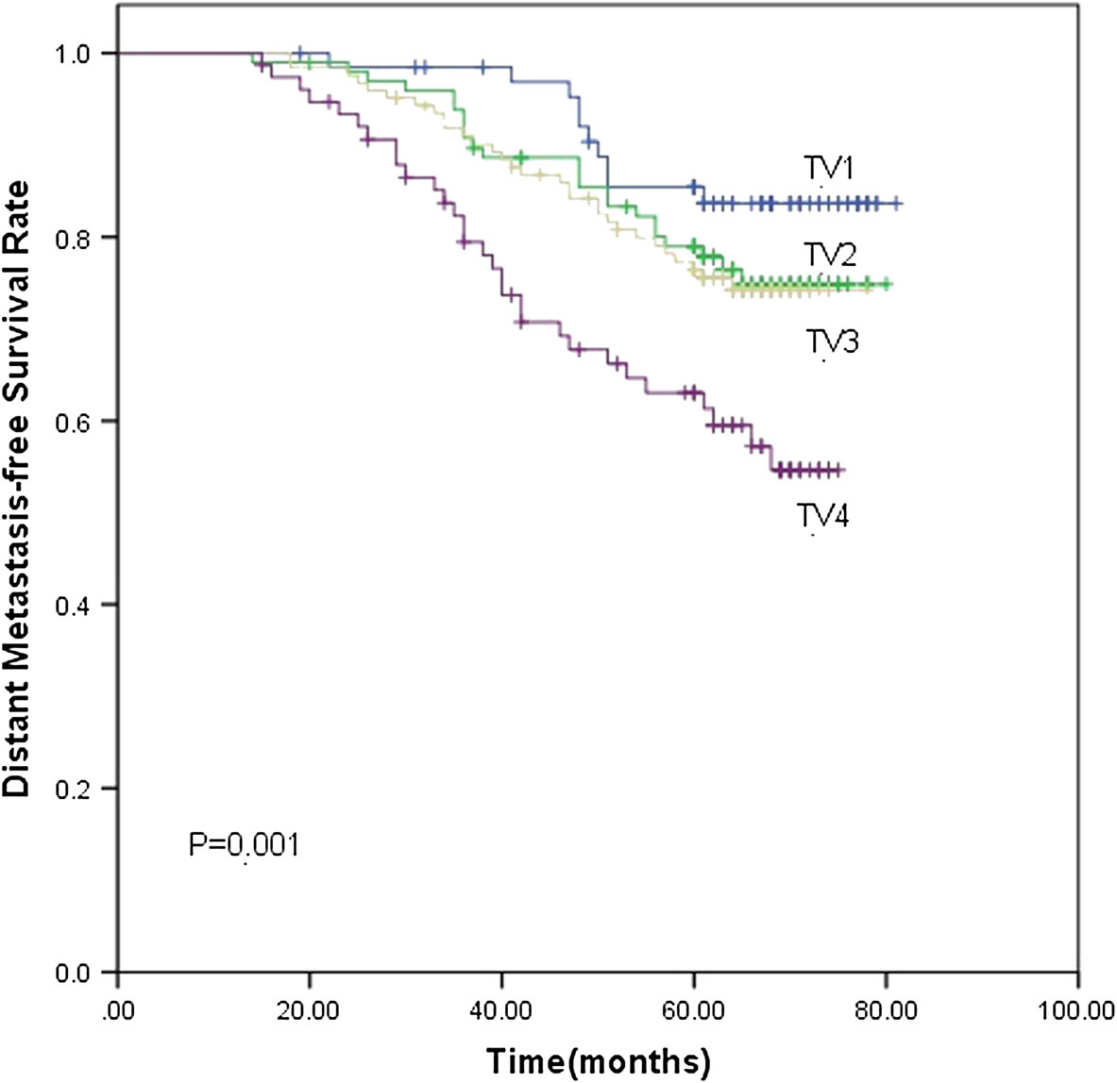
**Kaplan-Meier curves showing the 5-year DMFS rate in TV1, TV2, TV3 and TV4 groups.** There was a significant difference between the groups (p=0.001). The statistical analyses between each pair of groups were as follows: TV1 vs. TV2, *p*=0.216; TV1 vs. TV3, *p*=0.142; TV1 vs. TV4, *p*=0.000; TV2 vs. TV3, *p*=0.802; TV2 vs. TV4, *p*=0.006; and TV3 vs. TV4, *p*=0.009.

**Figure 3:**
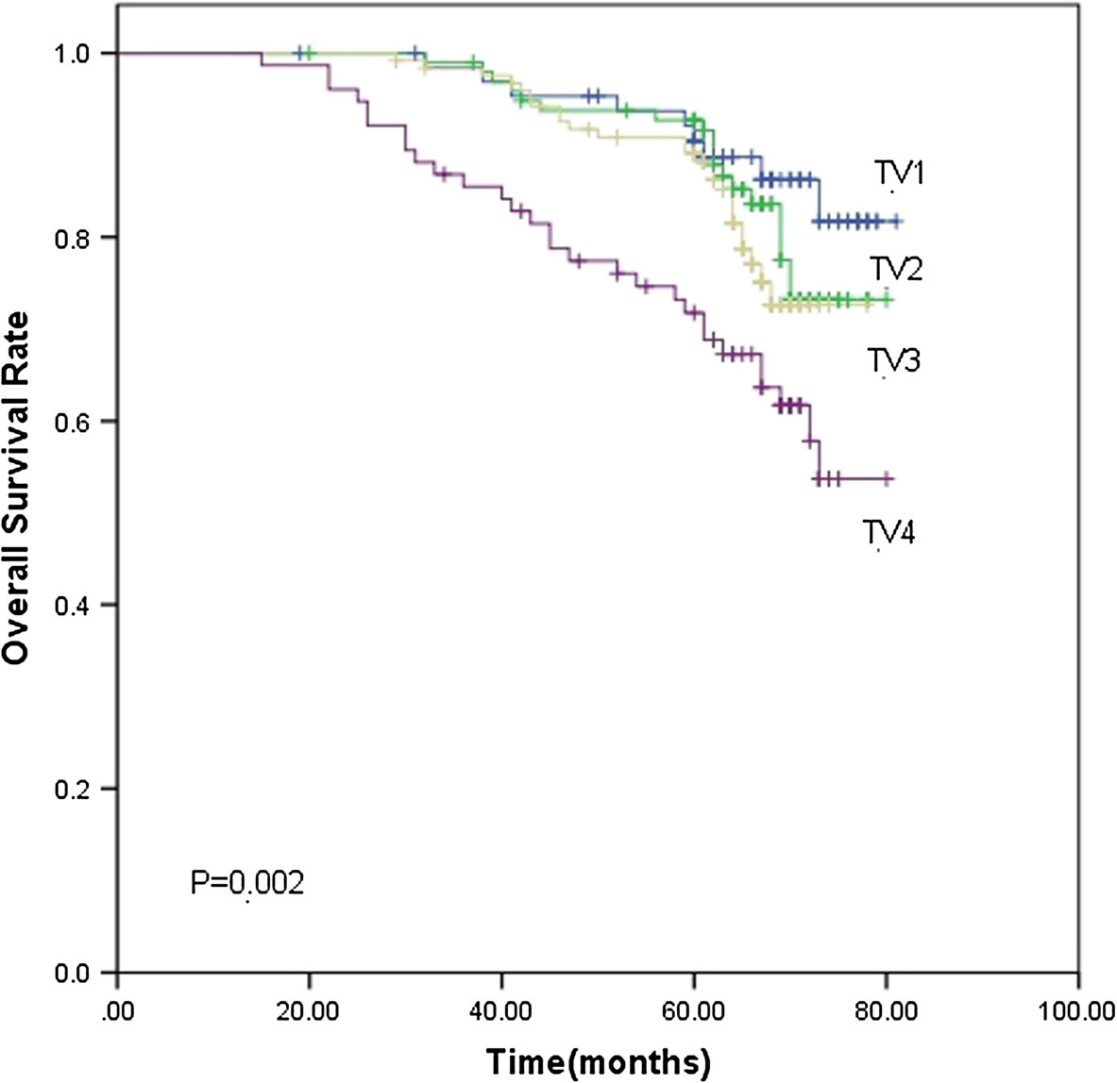
**Kaplan-Meier curves showing the 5-year OS rate in TV1, TV2, TV3 and TV4 groups.** There was a significant difference between the groups (*p*=0.002). The statistical analyses between each pair of groups were as follows: TV1 vs. TV2, *p*=0.387; TV1 vs. TV3, *p*=0.150; TV1 vs. TV4, *p*=0.001; TV2 vs. TV3, *p*=0.470; TV2 vs. TV4, *p*=0.005; and TV3 vs. TV4, *p*=0.019.

**Figure 4:**
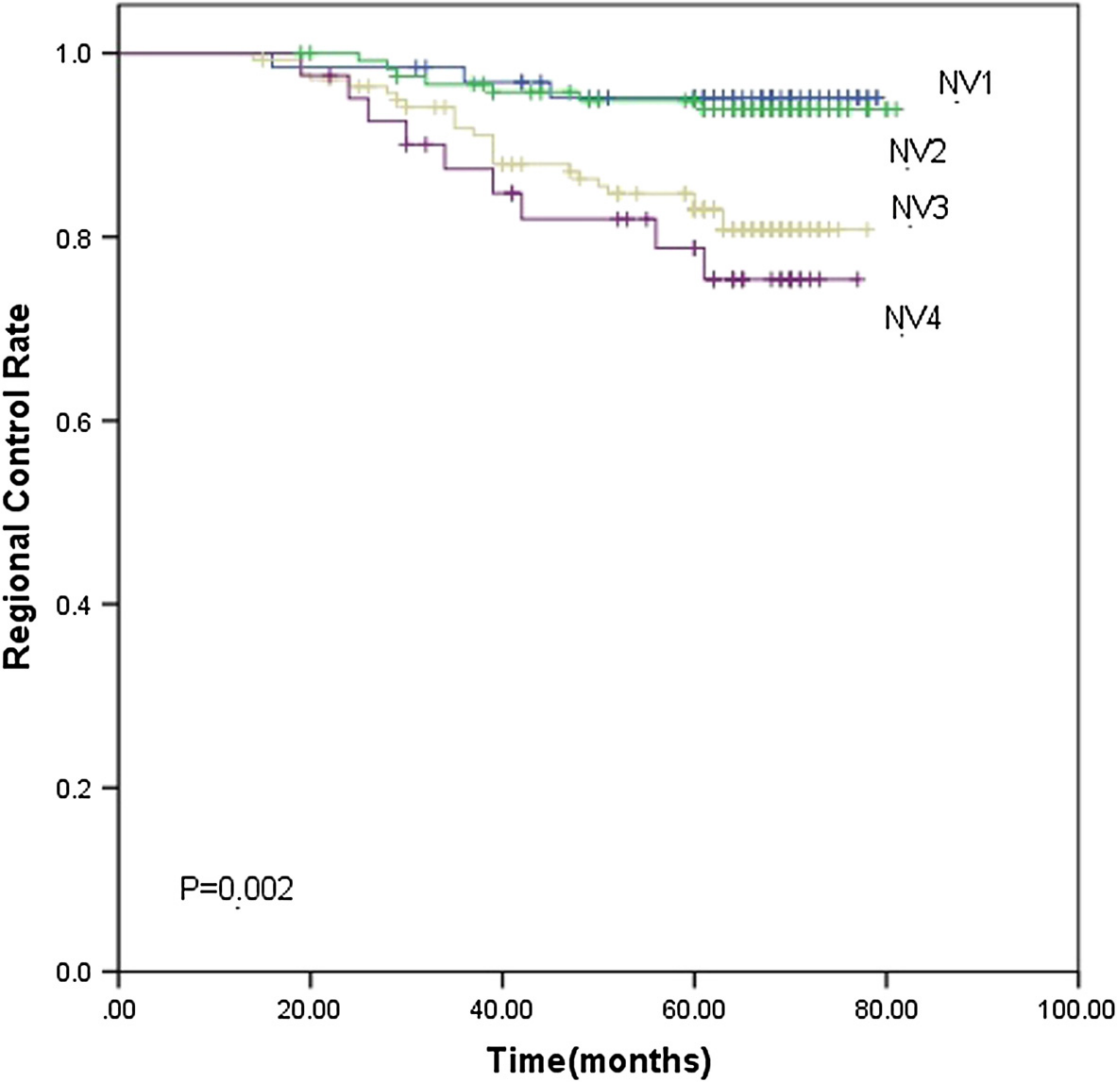
**Kaplan-Meier curves showing the 5-year RC rate in NV1, NV2, NV3 and NV4 groups.** There was a significant difference between the groups (*p*=0.002). The statistical analyses between each pair of groups were as follows: NV1 vs. NV2, *p*=0.750; NV1 vs. NV3, *p*=0.014; NV1 vs. NV4, *p*=0.005; NV2 vs. NV3, *p*=0.004; NV2 vs. NV4, *p*=0.001; and NV3 vs. NV4, *p*=0.439.

**Figure 5:**
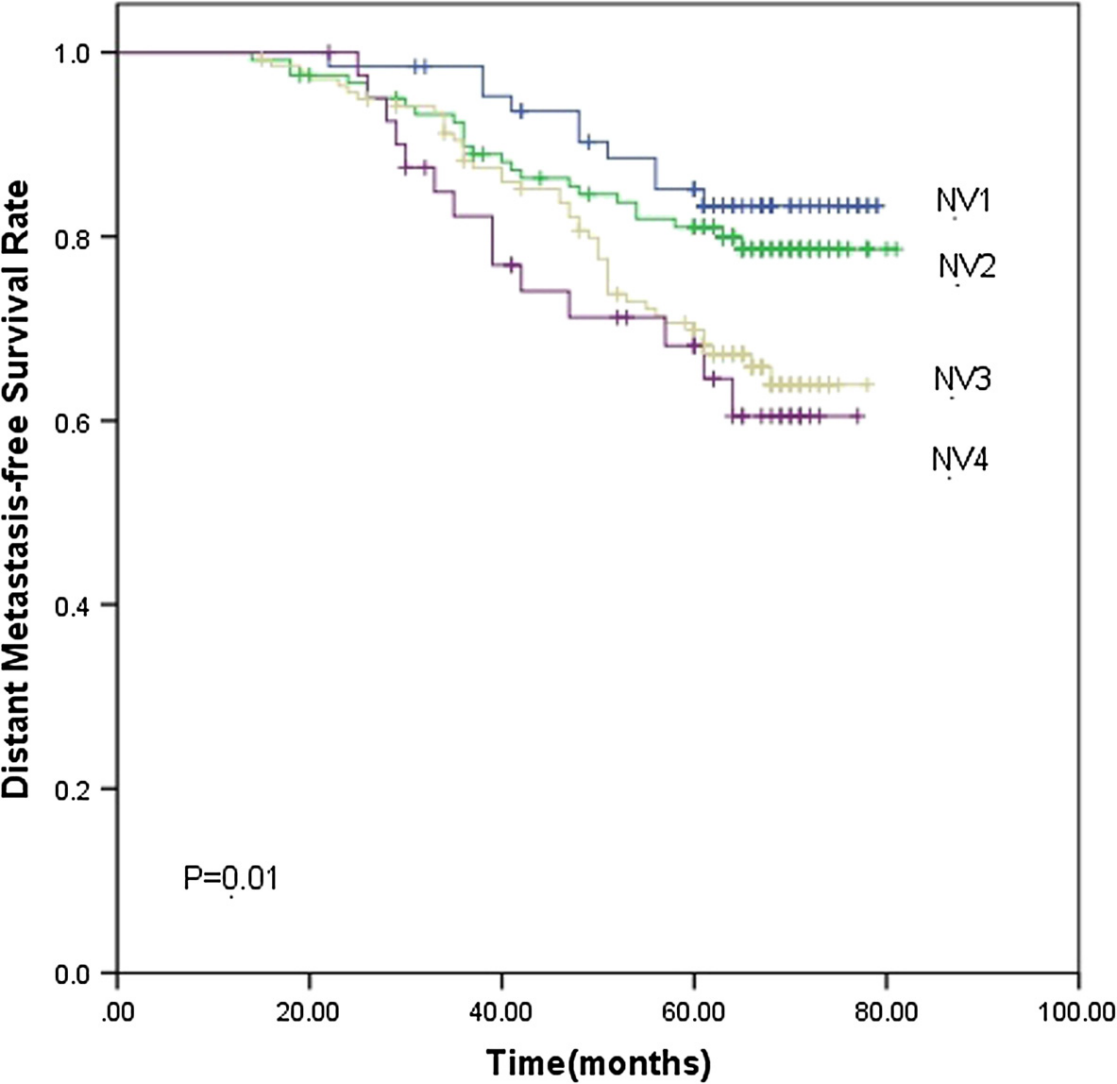
**Kaplan-Meier curves showing the 5-year DMFS rate in NV1, PNV2, NV3 and NV4 groups.** There was a significant difference between the groups (*p*=0.01). The statistical analyses between each pair of groups were as follows: NV1 vs. NV2, *p*=0.435; NV1 vs. NV3, *p*=0.012; NV1 vs. NV4, *p*=0.010; NV2 vs. NV3, *p*=0.027; NV2 vs. NV4, *p*=0.034; and NV3 vs. NV4, *p*=0.548.

**Figure 6:**
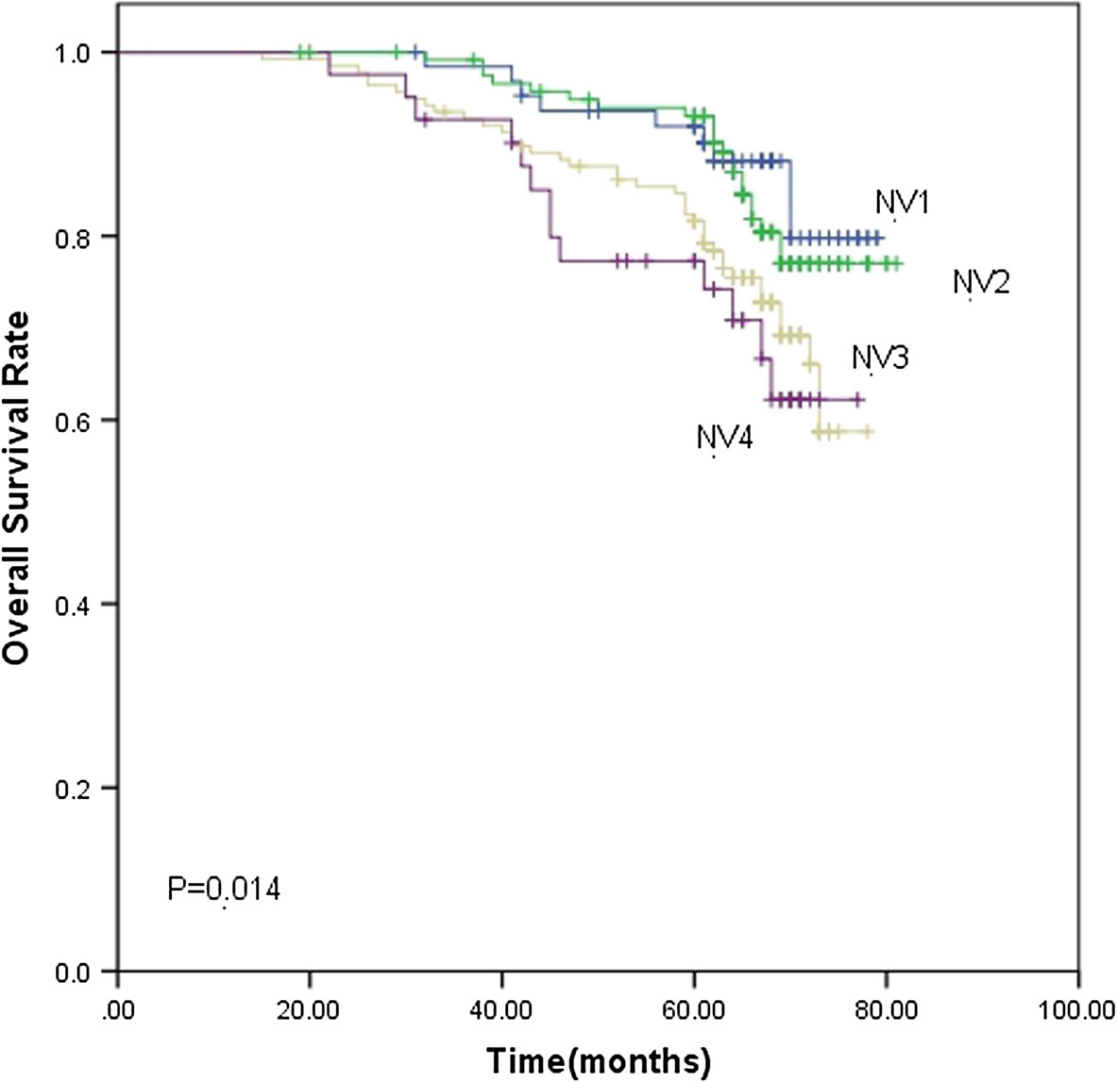
**Kaplan-Meier curves showing the 5-year OS rate in NV1, NV2, NV3 and NV4 groups.** There was a significant difference between the groups (*p*=0.014). The statistical analyses between each two groups were as follows: NV1 vs. NV2, *p*=0.687; NV1 vs. NV3, *p*=0.026; NV1 vs. NV4, *p*=0.023; NV2 vs. NV3, *p*=0.023; NV2 vs. NV4, *p*=0.026; and NV3 vs. NV4, *p*=0.490.

Based on the subgroups of primary tumor and nodal volume defined previously, the 5-year LC rate for TV1, TV2, TV3 and TV4 were 98.5%, 96.9%, 91.4% and 90.5% (*p*<0.001), respectively. The 5-year DMFS rate were 83.7%, 77.8%, 76.4% and 61.3%, respectively (*p*=0.001). TV4 had significantly lower LC (Figure 1) and DMFS (Figure 2) rate than the other three groups. As for the 5-year OS rate, they were 83.7%, 77.8%, 76.4% and 61.3% in the TV1, TV2, TV3 and TV4 groups (*p*=0.002), respectively. The 5-year OS rate for TV3 and TV4 was significantly lower than the other two groups ((Figure 3). The 5-year RC rate for the NV1, NV2, NV3 and NV4 groups were 95.1%, 93.9%, 83.1% and 75.4% (*p*<0.001), respectively. The 5-year DMFS rate for these groups were 83.3%, 80.0%, 69.8% and 64.0%, respectively, and the 5-year OS rate were 90.2%, 88.2%, 81.6% and 74.2%, respectively (*p*<0.001; *p*<0.001). The 5-year RC (Figure 4), DMFS (Figure 5) and OS (Figure 6) rate were significantly lower in NV3 and NV4 groups when compared to NV1 and NV2 groups.

### Multivariate analysis

Primary tumor volume, N stage and chemotherapy were independent prognostic factors in this study. TV>40 ml was an adverse prognostic factor for the 5-year LRC rate (Table 3). N stage (N2/N3) was an adverse prognostic factor for the 5-year DMFS and OS rate (Tables 4, 5). Chemotherapy improved both the 5-year DMFS (Table 4) and OS (Table 5) rate. Primary nodal volume, T stage, sex and year had no statistical significance in multivariate analysis (Tables 3, 4, 5).

**Table 3:** Multivariate analysis predicting 5-year LRC rate

**Prognostic factors**	**Hazard ratio**	**95% CI**	***P *value**
Sex	1.240	0.707-2.177	0.453
Year	1.104	0.699-1.743	0.671
TV	2.454	1.407-4.280	0.002
NV	1.074	0.632-1.826	0.793
T stage	1.175	0.711-1.942	0.528
N stage	1.231	0.774-1.956	0.380
Chemotherapy	1.366	0.833-2.240	0.216

**Table 4:** Multivariate analysis predicting 5-year DMFS rate

**Prognostic factors**	**Hazard ratio**	**95% CI**	***P *value**
Sex	1.116	0.680-1.832	0.665
Year	0.893	0.588-1.356	0.596
TV	1.549	0.928-2.584	0.094
NV	1.457	0.903-2.352	0.123
T stage	1.433	0.896-2.293	0.133
N stage	1.600	1.048-2.442	0.030
Chemotherapy	1.813	1.172-2.805	0.008

**Table 5:** Multivariate analysis predicting 5-year OS rate

**Prognostic factors**	**Hazard ratio**	**95% CI**	***P *value**
Sex	1.360	0.772-2.396	0.288
Year	0.938	0.599-1.471	0.781
TV	1.393	0.802-2.417	0.239
NV	1.480	0.883-2.482	0.137
T stage	1.670	0.997-2.796	0.239
N stage	1.662	1.048-2.635	0.031
Chemotherapy	2.360	1.498-3.717	0.000

## Discussion

We evaluated the prognostic significance of the primary and nodal tumor volume in NPC patients treated with IMRT based on CT and MRI images. Univariate and multivariate analysis showed that primary tumor volume was a new prognostic factor for survival rate. A primary tumor volume of 40 ml may be the cut-off value. N stage and chemotherapy remain important prognostic factors for the 5-year DMFS and OS rate. Primary nodal volume could predict the prognosis in univariate analysis, but had no statistical significance in multivariate analysis.

Our univariate analysis showed that a TV>40 ml and NV≥10 ml indicated the worse 5-year LC, RC, DMFS and OS rate. However, only a TV >40 ml was an independent prognostic factor for the 5-year LRC rate in multivariate analysis. Primary nodal volume had no statistical effect on the 5-year LRC rate. Neither primary tumor volume nor primary nodal volume was significantly different in terms of the 5-year DMFS or OS rate. Chen [6] revealed that the 5-year local failure-free rate (LFFR) for V1 (<15.65 ml), V2 (15.65-24.25 ml), V3 (24.25-50.55 ml) and V4 (>50.55 ml) were 96.2%, 93.3%, 88.2% and 77.3%, respectively, in 112 NPC patients treated with IMRT. TV4 had a significantly lower LFFR and OS rate than the others. Chua [7] analyzed the prognostic significance of the volume of the primary tumor and lymph nodes in 290 NPC patients. The results showed that a large variation in tumor volume was present in different T stages of nasopharyngeal carcinoma, and primary tumor volume (>60 ml) represented an independent adverse prognostic factor for LC rate. A large nodal tumor volume (>30 ml) was associated with significantly higher distant failure and lower disease-specific survival rate and was significantly different in multivariate analysis. Kim [8] also showed that a large primary tumor volume (>30 ml) was associated with a significantly lower RC rate in 60 NPC patients. Additionally, nodal volume (>5 ml) was associated with significantly lower regional control and lower disease-specific survival rate. However, these three studies all used CT images to delineate tumor volume. In a recent study, Sarisahin [9] delineated tumor volumes based on both CT and MRI, and evaluated the prognosis of 56 NPC patients. The primary tumor volume (>60 ml) was found to be significant predictor for LC, DFS and DMFS in univariate analysis. Together with these previous studies, the findings in our study strongly support the prognostic value of primary tumor volume in predicting the 5-year LRC rate for NPC. However, the best cut-off of primary tumor volume remains to be determined. In our study, primary tumor volume had a statistical effect in univariate analyses for predicting the 5-year LC rate, and a PV of 40 ml may be the cut-off value. Also, the multivariate analysis comes to the same conclusion. More high-quality studies are needed to confirm this. In addition, our univariate analysis showed the primary nodal volume could predict the 5-year RC, DMFS and OS. But the multivariate analysis found no statistical difference. Only limited number of studies [7,8,10] discussed on the relationship between primary nodal volume and prognosis, and the cut-off values were different among these studies. The variations could be caused by different reference images used in target delineation. Most studies used CT images to delineate the primary tumor, but we used both CT and MRI images to acquire tumor volume, a more accurate method than CT alone. Several studies showed MRI defined the extent of nasopharyngeal disease better than CT did by its superior capability in demonstrating primary soft tissue invasion and subtle intracranial invasion [11]. The registration of CT and MRI images may be essential in NPC treatment planning, because the complementary information contained in the two modalities could provide more accurate target definition. In our present study, we did not compare the difference between CT and MRI defined target volumes, but several studies showed that CT and MRI images might lead to different target volume in head and neck carcinoma. For NPC, Emami [12] showed that MRI provided larger target volumes compared with CT. But Rasch [13] found CT displayed 30% larger volumes than MRI in advanced head-and-neck cancer patients. In addition to different imaging modality, the target volumes were also susceptible to interobserver variations [14]. Different oncologists may perceive different criteria in delineation. The interobserver variation always existed in target delineation, but different modality might cause interobservier variation in different degree. Chung [11] found MRI targets had smaller interobserver differences. So, owing to the improved visualization in MRI, it might be an important method to decrease the interobserver difference in NPC patients which was used in our study. Beyond that, there was still no consensus in the method for measuring volume. Diameter-based volume, summation of areas and a volume algorithm were all used in different studies. So, it was still hard to evaluate the difference between MRI and CT based target volume in NPC, further studies were needed to resolve these issues.

In the univariate analysis of our study, T and N stage had no significant effect on the 5-year LC and RC rate, but they affected the 5-year DMFS and OS rate significantly. In multivariate analysis, it was revealed that N stage and chemotherapy were the important prognostic factors for the 5-year DMFS and OS trate. However, T stage was not predictive for these endpoints. This result was consistent with two recent studies. Han [15] reported that T stage was not an independent prognostic factor for LC and OS in 305 patients with NPC treated with IMRT. Bilgin [3] found that T and N stage had no significant effect on LC or RC. N stage and response to treatment were the most important independent predictors of OS in their study. According to these findings, we proposed that the current TNM staging system should include the tumor volume as an important staging factor that could define the real and three-dimensional tumor burden. A larger primary tumor and nodal volume mean a heavier tumor load, usually resulting in hypoxia, radiation resistance and poor rates of tumor control. Given the fact that tumor volume may have broad variation even in the same T classification, it is conceivable that in the current study, primary tumor volume had more predictive power than the T stage for LC. In our study, N stage remained important for predicting DMFS and OS, but it was less effective in predicting RC than primary nodal volume, although none of these were statistically significant.

PET-CT has an advantage in indicating the biological margins of tumors, which may have an important role in defining tumor volume. The current study did not include PET-CT which warrants a future study on the prognostic role of primary tumor volume based PET-CT images for NPC patients. The cut-off value for tumor and nodal volume was another issue that needs further discussion. This finding might be explained by inter-observer and inter-institution variation in target delineation and volume calculation, which may influence the predictive value of primary tumor and nodal volume. The specific correlation between the tumor volume and treatment strategy was not analyzed in this study. Chen [16] considered that large primary tumor volume would require more aggressive treatment. Lee [17] reported that OS was better after ≥4 cycles of chemotherapy than after fewer than 4 cycles for a large primary tumor and retropharyngeal lymph node volume (≥13 ml). A large randomized controlled study is needed to address whether intensive chemotherapy would alter the prognosis of NPC with large primary tumor volume.

## Conclusions

Based on survival analysis of 365 cases of NPC consecutively treated in our center from 2003–2005, we found that primary tumor volume could be used as a prognostic factor for NPC. This factor was more effective than T classification in predicting LC. Primary nodal volume had greater potential to predict RC than N stage but was not statistically significant. We suggest that primary tumor volume should be included in NPC staging and considered an important factor for predicting treatment outcomes.

## Competing interests

There are no Competing interest to declare.

## Authors’ contributions

MF performed clinical data analyses, assisted in patient treatment and wrote the manuscript. WDW, ZXF and BYF participated in the data analysis and organizing follow-up examinations. WDW also helped to approve the treatment plan. JL helped to formulation the radiation planning and the analysed the data of tumor volumes. SCZ carried out the interpretation of CT and MRI images and analysed the tumor volumes data. JYL approved treatment plans, supervised patient treatment and helped to finalize the manuscript. All authors read and approved the final manuscript.
